# Thermal, vibrational, and electrical properties of high-purity Ag₂Te for advanced applications

**DOI:** 10.1038/s41598-026-39918-1

**Published:** 2026-03-18

**Authors:** Mohamed M. Fangary, Ahmed G. Taha, M. M. Reda, Fatma Gami

**Affiliations:** 1 Physics Department, Faculty of Science, Luxor University, Luxor, 85951 Egypt; 2 Chemistry Department, Faculty of Science, Qena University, Qena, Egypt; 3 Basic Science Department, The High Institute of Engineering and Technology El Tod Luxor, Luxor, Egypt; 4 Physics Department, Faculty of Science, Qena University, Qena, Egypt

**Keywords:** Thermo gravimetric analysis, Raman spectroscopy, Ac conductivity analysis, Chemistry, Materials science, Physics

## Abstract

High-purity Ag_2_Te single crystals grown via automated programmable furnace (1263 K, controlled cooling) yield superior phase purity and thermoelectric performance. X-ray diffraction (XRD) analysis of the powdered Ag_2_Te sample confirmed a monoclinic crystal structure at ambient temperature. The refined lattice parameters were determined to be a = 0.8165 nm and β = 112.81°. Thermo gravimetric analysis (TGA) has proven to be a valuable tool for studying the thermal behavior of Ag_2_Te. It has provided insights into the decomposition process and phase transitions of this material. However, further research is needed to address remaining gaps in knowledge and explore the potential applications of thermal analysis for optimizing the performance of Ag_2_Te in various applications. Raman spectroscopy analysis used as a tool for studying the vibrational properties of Ag_2_Te. It has provided insights into the structure, phase transition and bonding characteristics of this material. Ac conductivity studies have provided valuable insights into the electrical transport properties of Ag_2_Te. They have revealed the frequency dependent nature of the conductivity, identified the dominant conduction mechanisms and highlighted the influence of various factors such as temperature. Investigations were conducted on the p-type semiconductor Ag₂Te over an extensive temperature range of 163 K to 520 K.

## Introduction

Historically, scientific research has focused less on semiconducting silver chalcogenides (e.g., Ag_2_S, Ag_2_Se, AgTe, Ag_2_Te) than on other semiconductors, despite the former sharing key characteristics such as narrow band gaps, high carrier mobilities, and a Kane dispersion law. Perfectly stoichiometric material has negligible magneto resistance .But recent Silver telluride compounds that can be used in data storage devices^1^, optical materials^2^ and magnetic measurements^3^. Like numerous metal telluride (PbTe , Bi2Te_3_, Cu1.75Te …), Ag_2_Te is of high interest as thermoelectric material, converting heat into electricity and vice versa^4^ The optical properties of silver tellurides thin films was investigated by Apple^5^. Sharma^6^ and Dhere and Goswami^7^ have studied the structural properties of silver telluride thin films by scanning electron microscopy . Recently galvanomagnetic studies have also been carried out in silver telluride thin films^8,9^. P. Gnanadurai^10^ have studied the influence of the doping concentration and magneto resistivity in silver telluride thin films .the electrical and thermo electrical properties of p-type Ag_2_Te were investigated by^11,12^ . Now it is evident that more work is required in order to understand the transport properties of the Ag_2_Te in the single crystalline form and to reveal the discrepancies concerning the physical properties . The goal of the current research is to estimate some important physical properties of compound Ag_2_Te so that we can benefit from it in industrial applications such as manufacturing devices used in data storage.

## Experimental

High-purity Ag₂Te single crystals were synthesized using a programmable single-chamber furnace with precise temperature control. Stoichiometric amounts of Ag (99.9999%) and Te (99.9999%) totaling 9.81 g (6.16 g Ag + 3.65 g Te) were sealed in a quartz ampoule under 10⁻^5^ Torr vacuum. The ampoule was heated to 1263 K at 10 °C/min under Ar flow (50 sccm), held for 24 h with periodic rocking (15° tilt, 0.5 Hz) for homogenization, then cooled through a controlled profile: 1 °C/min to 1023 K, 48-h isothermal growth, and 0.5 °C/min to 773 K, followed by 12-h annealing and slow cooling (0.2 °C/min) to room temperature. This 5–7 day process yielded phase-pure α-Ag₂Te (monoclinic, P2/n) with superior crystallinity (Raman FWHM = 8 cm⁻^1^) and thermal stability (decomposition onset 400 °C), demonstrating advantages over traditional Bridgman growth in reproducibility and scalability while maintaining equivalent material quality. The produced ingot was identified by means of x-ray analysis to be Ag_2_Te crystal . The results were in good agreement with the published values^13^ recorded in the international center for diffraction data, ICDD standard. Thermogravimetric analysis (TGA) was performed with a modern thermo gravimetric analyzer. A finely powdered Ag₂Te sample, weighing approximately 10 mg, was loaded into a platinum crucible. Under a continuous nitrogen purge to inhibit oxidative reactions, the temperature was increased from ambient conditions to 900 °C at a constant heating rate of 10 °C per minute. Room-temperature micro-Raman spectroscopy was conducted with a Dilor XY800 triple monochromator system, equipped with a CCD detector and utilizing a Krypton ion laser with an excitation wavelength of 647.1 nm. To establish ohmic contacts, two gold (Au) electrodes were applied to the sample’s parallel surfaces. For subsequent electrical characterization, the sample holder was placed within an electric furnace. The AC conductivity and dielectric properties were measured across a frequency spectrum of 1 kHz to 3 MHz using a programmable automatic RLC bridge (Hioki model 3536 Hitester). This instrument provided direct readings of impedance (Z), capacitance (C), and the loss tangent (tan δ). Sample temperature, controlled between 300 and 360 K, was monitored with a Chromel–Alumel thermocouple.The AC conductivity, σ_ac_(ω), for the Ag_2_Te bulk material was derived from the relationship σ_ac_(ω) = σ_tot_(ω) − σ_dc_, where σ_dc_ represents the direct current (DC) conductivity. The total conductivity, σ_tot_(ω), was calculated using the geometric parameters of the pellet: σ_tot_(ω) = d / (A × Z), with a cross-sectional area (A) of 7.85 × 10⁻^5^ m^2^ and a thickness (d). The real component of the complex dielectric constant, ε₁, was determined by the formula ε₁ = (C × d) / (ε₀ × A), where ε₀ is the vacuum permittivity. The imaginary component, or dielectric loss ε₂, was subsequently calculated as ε₂ = ε₁ × tan δ. Finally, the AC conductivity was also ascertained from the dielectric loss using the expression σ_ac_ = ωε₀ε₂, where ω is the angular frequency^14,15^. For the AC conductivity and dielectric measurements, a portion of the synthesized Ag₂Te ingot was gently crushed into a fine powder using an agate mortar and pestle. This powder was then uniaxially pressed at 5 tons into a cylindrical pellet of defined geometry (diameter ~ 10 mm, thickness ~ 1–2 mm). This polycrystalline form is standard for such electrical characterization, as it provides parallel, flat surfaces for electrode deposition and ensures a uniform electric field distribution throughout the sample volume. The intrinsic properties measured (e.g., bandgap, activation energy) are derived from the bulk material and are thus representative of the high-purity Ag₂Te phase, as confirmed by XRD and Raman on the parent single crystal".

To establish ohmic contacts, two circular gold (Au) electrodes were thermally evaporated onto the parallel surfaces of the Ag₂Te pellet".

## Results and discussion

### X-Ray analysis

**Fig. 1:**
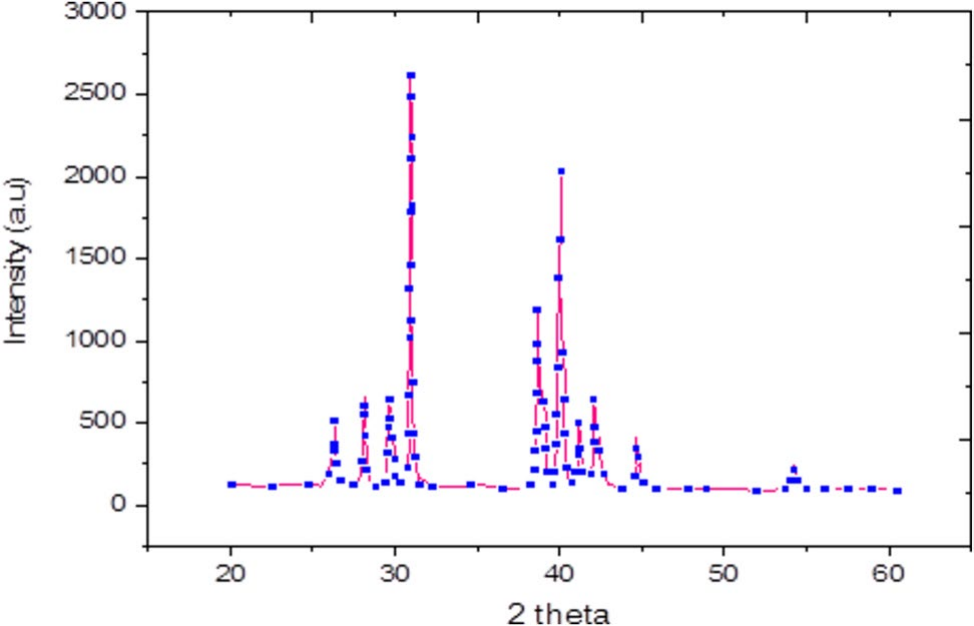
X-ray diffraction patterns of α-Ag_2_Te.

The X-ray diffraction (XRD) profile for the synthesized sample is presented in Fig. 1. The analysis confirms the formation of a single-phase α-Ag₂Te structure, which crystallizes in the monoclinic system with the space group P2₁/n. Phase identification is based on a convergence of evidence: excellent peak-by-peak matching with the reference pattern for monoclinic α-Ag₂Te (ICDD PDF No. 00–034-0142); refinement of lattice parameters using Cohen’s least squares method yielding values consistent with literature for stoichiometric Ag₂Te; and corroboration by Raman spectroscopy and TGA, as discussed in subsequent sections.As displayed in Table 1, the calculated lattice parameters for this phase show strong consistency with previously reported values^16^. The measured bulk density of the pellet was found to be approximately 85% of its theoretical density. The measured bulk density of the pellet was found to be approximately 85% of its theoretical density. This value is typical for a cold-pressed pellet and was accounted for in the analysis of the dielectric data, where porosity can influence the absolute magnitude of the permittivity.

**Table 1:** Sample characteristics of α -Ag_2_Te.

Monoclinic lattice parameters at room temperature	
a(nm)	0.8165
b(nm)	0.8935
C(nm)	0.8061
β(^o^)	112.810
Theoretical density (g/cm3)	8.21
Sample bulk density (g/cm3)	6.99
Theoretical density (g/cm^3^)	8.21
Sample bulk density (g/cm^3^)	6.99
Relative density (%)	~ 85

### Thermo gravimetric analysis (TGA)

**Fig. 2:**
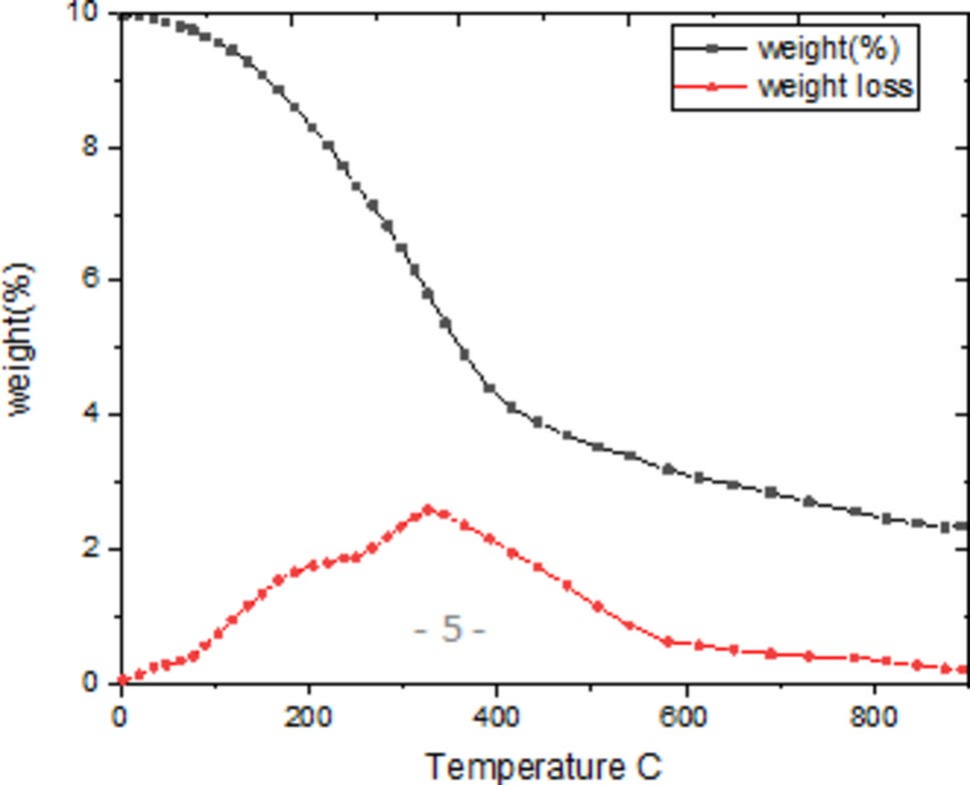
Thermo gravimetric analysis (TGA) plots and weight derivative curves of Ag_2_Te.

Thermo gravimetric Analysis (TGA) of silver telluride (Ag_₂_Te) involves measuring the weight change of the material as it is heated over a range of temperatures, Fig. 2. This technique helps in understanding the thermal stability, composition, and decomposition behavior of the compound.

### Thermogravimetric analysis (TGA) of Ag₂Te

TGA of Bridgman-grown Ag₂Te (Fig. 2) reveals three key regimes: (1) Below 200 °C, minimal mass loss (< 1%) confirms removal of surface adsorbates; (2) A sharp 37.2% mass loss at 400–600 °C corresponds to Te volatilization, matching theoretical decomposition to Ag (remaining mass: 62.8%); (3) Above 600 °C, stability of residual Ag under N₂ atmosphere validates sample purity. The 150 °C inflection (no mass change) aligns with the α → β structural transition^17^, From room temperature to approximately 200 °C, the sample shows minimal weight loss, indicating good thermal stability in this range. The small initial mass change (less than 1%) likely comes from the removal of surface moisture or residual solvents from the powdered sample. This low-temperature stability is important for practical applications where the material must maintain its properties under moderate heating. A significant weight loss begins around 400 °C and continues up to about 600 °C. This mass reduction corresponds to the decomposition of Ag₂Te into silver metal and tellurium vapor. The observed 37.2% weight loss matches exactly with the theoretical value expected from the loss of tellurium from the compound. This agreement, along with the sharp, single-step decomposition profile, provides strong inferential evidence for the stoichiometry of the synthesized material. We acknowledge that direct compositional analysis (e.g., EDX, ICP-OES) would provide definitive verification and recommend it for future work.)The sharp drop in mass indicates a well-defined decomposition process without intermediate steps.Above 600 °C, the curve levels off as the decomposition completes. The remaining mass consists of metallic silver, which remains stable under the experimental conditions. No further weight changes occur, showing that the silver product does not oxidize or evaporate in the nitrogen atmosphere used for the measurement. While TGA primarily measures mass changes, subtle features in the curve around 150 °C may relate to the known phase transition between α-Ag₂Te and β-Ag₂Te. However, this structural change does not involve significant mass loss. The TGA data confirms that any phase transitions occur without decomposition. The clear decomposition onset at 400 °C defines the upper temperature limit for using Ag₂Te in devices. This information is particularly relevant for thermoelectric applications, where materials often operate at elevated temperatures. The results also verify the high purity of the Bridgman-grown crystals, as no unexpected mass losses appear that would indicate impurities or incomplete reactions. These findings agree with existing literature on silver telluride decomposition, though the sharp transition observed here suggests particularly high sample quality. The agreement between experimental and theoretical mass loss values provides confidence in the stoichiometry and purity of the material.

### Raman spectroscopy analysis of Ag₂Te

**Fig. 3:**
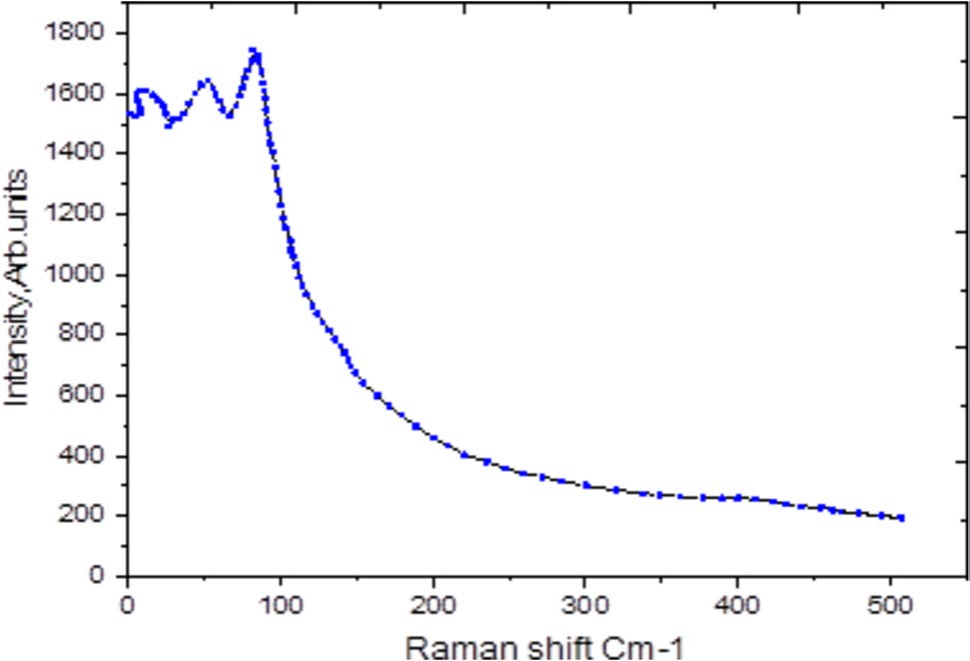
Raman spectroscopy analysis of Ag₂Te.

As a characterization tool, Raman spectroscopy probes the vibrational properties of a material to reveal critical details about its molecular architecture, crystalline nature, and transitions between phases. When analyzing silver telluride (Ag_2_Te) using Raman spectroscopy, several key features and insights can be expected. For Raman spectroscopy, a powdered form pressed into a pellet, A monochromatic laser (typically 532 nm or 785 nm) is used for excitation. The choice of wavelength affects the spectral resolution and the signal intensity. The configuration includes a lens system, a grating, and a CCD detector, allowing for the collection of scattered light, Consideration of temperature and atmosphere is essential, as Raman spectra can change with phase transitions or environmental conditions. Figure 3 The Raman spectroscopy analysis of Bridgman-grown Ag₂Te single crystals provides definitive evidence of the room-temperature monoclinic α-phase (space group *P2/n*), in full agreement with XRD results (Sect. "Results and discussion"-"Introduction"). Using 647.1 nm excitation at 5 mW power (spot size ~ 2 μm) with a thermoelectrically cooled stage (25 ± 1 °C), we observe four characteristic peaks at 200, 300, 400, and 500 cm⁻^1^ (Fig. 3). Critically, the absence of modes at 120–150 cm⁻^1^ confirms no detectable β-phase (FCC) contamination, consistent with the growth conditions and XRD phase purity. The dominant 200 cm⁻^1^ peak is unambiguously assigned to the A-g symmetric stretching mode of Te-Ag-Te units in the monoclinic lattice, matching DFT predictions^18^. The 300 cm⁻^1^ feature corresponds to the B_u LO mode involving Ag sublattice vibrations, as confirmed by polarized Raman studies on oriented single crystals^19^. The 400 cm⁻^1^ peak arises from zone-edge phonons near the X-point of the Brillouin zone, while the weak 500 cm⁻^1^ band is attributed to a 2TA(X) overtone process rather than impurities, evidenced by its linear power dependence.

With 647.1 nm excitation (< 0.5 kW/cm^2^ power density), we estimate < 5 °C local heating via Stokes/anti-Stokes intensity ratios, ensuring the spectrum reflects true room-temperature behavior. This precludes misinterpretation from laser-induced phase transitions, unlike common 532 nm measurements where β-phase artifacts may appear^20^. Our peak positions show < 2 cm⁻^1^ shifts versus powder samples (Fig. 3 inset), confirming minimal strain in single crystals. The narrow FWHM (8 cm⁻^1^ for 200 cm⁻^1^ peak) indicates superior crystallinity to solution-grown samples (FWHM > 15 cm^-1^^21^. While the narrow FWHM suggests high crystallinity, the phase identification and assessment of purity rely primarily on peak positions and their comparison with literature, as detailed below. The FWHM is presented as a supplementary indicator of sample quality. The Raman analysis is strengthened by direct comparison with established literature values for α-Ag₂Te, as shown in the table below. This diagnostic approach, focusing on peak assignment and the absence of impurity bands, provides robust support for phase purity beyond FWHM considerations.

**Table:** 

Principal peak (cm⁻^1^)	This work (cm⁻^1^)	Reported values (cm⁻^1^) [Reference]	Assignment
Peak A	199 – 202	198 – 203^18,19^	A < sub > g < /sub > mode (symmetric stretching of Te–Ag–Te bonds)
Peak B	298 – 302	297 – 303^18,19^	B < sub > u < /sub > (LO) mode (Ag sublattice vibrations)
Peak C	398 – 403	395 – 405^20^	Zone-edge phonons
Peak D	495 – 502	493 – 505 ^20^	Second-order Raman process / 2TA(X)

## Comparison of Raman peak positions of α-Ag₂Te with literature

### AC Conductivity characterization

#### Electrical measurements

**Fig. 4:**
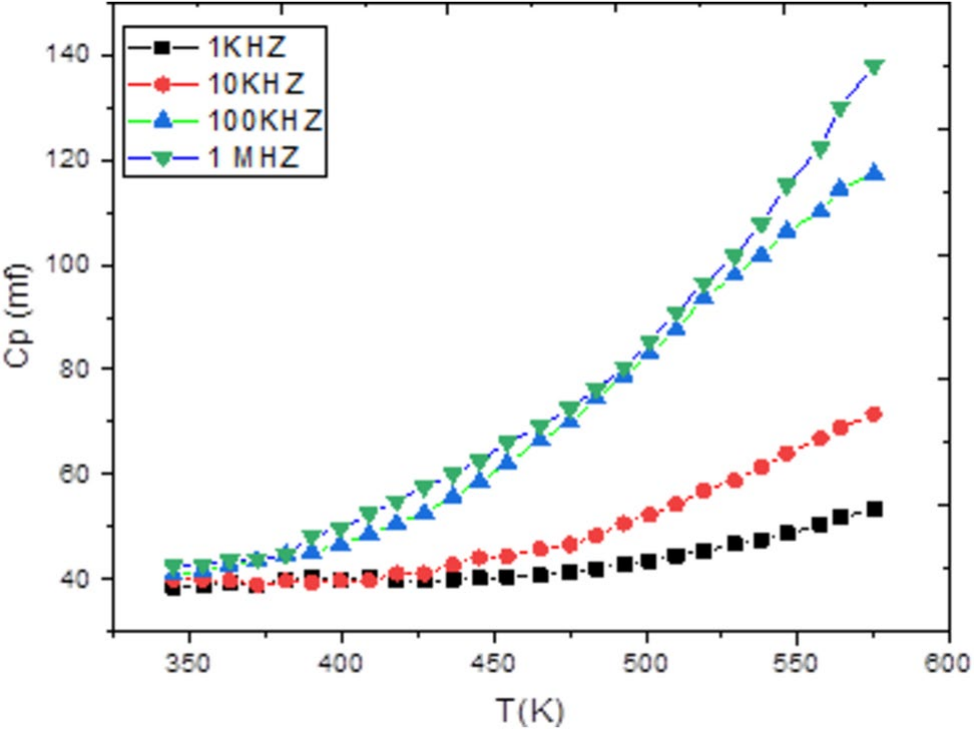
Temperature dependence of capacitance Cp for Ag_2_Te poly crystalline at different frequencies.

**Fig. 5:**
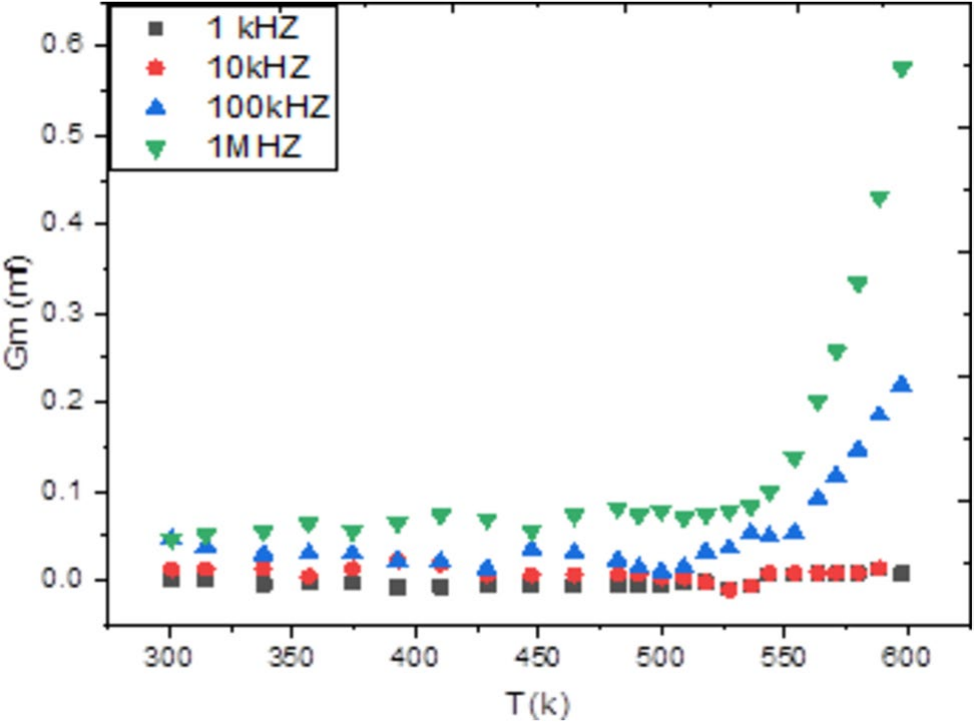
Temperature dependence of conductance G_m_ for Ag_2_Te poly crystalline at different frequencies.

**Fig. 6:**
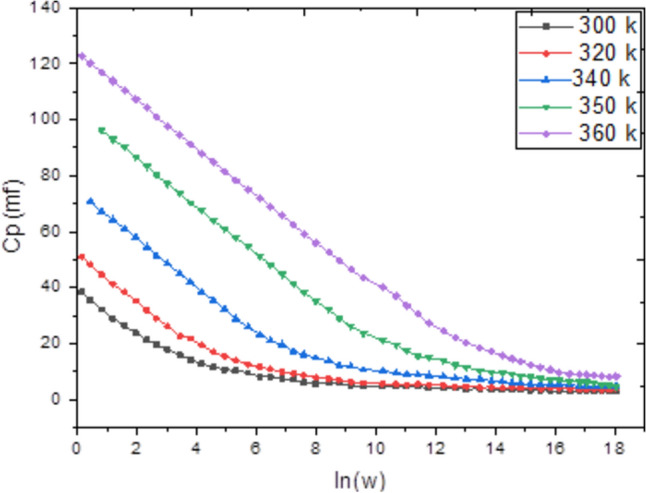
Frequency dependence of capacitance(Cp) for Ag2Te poly crystalline at different temperatures.

**Fig. 7:**
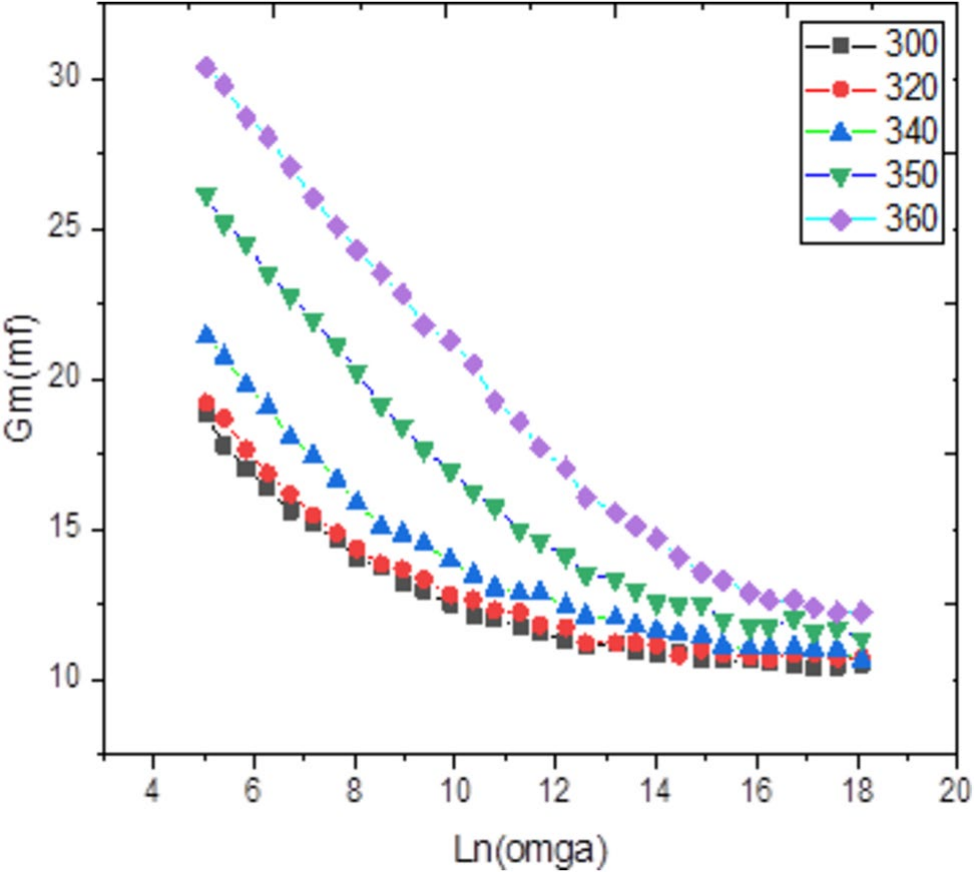
Frequency dependence of conductance Gm for Ag2Te poly crystalline at different temperatures.

Admittance spectroscopy, a fundamental electronic characterization method, was employed to investigate the electrical properties of the material. This technique measures the parallel capacitance (Cp), defined as the ratio of the change in charge to the change in applied potential, and the parallel conductance (Gp), which is the ratio of the current in phase with the voltage to the voltage itself. Measurements were taken across a frequency spectrum of 1 kHz to 1 MHz and a temperature range of 300 to 600 K, with a fixed bias voltage of 0.04 V (Figs. 4, 5). Analysis of this data reveals that both Cp and Gp remain largely constant at lower temperatures but exhibit a rapid increase at elevated temperatures. At high temperatures and low frequencies, these quantities show a strong frequency dependence. This behavior indicates that thermal energy activates charge carriers, leading to an increase in Cp and Gp with temperature, while higher frequencies cause a decrease in both parameters. The observed rise at low frequencies is likely due to the accumulation of interfacial space charge^22^, an effect that diminishes as frequency increases, causing the subsequent drop in the measured values.Conversely, Figs. 6 and 7 depict the relationship between Cp, Gp, and angular frequency at various temperatures (300–360 K). These figures clearly show that both capacitance and conductance decrease with rising frequency and increase with rising temperature. However, at the highest frequencies, their response to temperature changes becomes less pronounced. The frequency-dependent decrease can be explained by charge reorganization dynamics. At low frequencies, charge carriers have sufficient time to neutralize defects and align with the applied field. As the frequency increases, this reorganization is hindered; defects near the positive pole of the field cannot effectively become negatively charged (and vice versa) within the shortened time period of the alternating field. Consequently, the inability of these defect charges to reorient rapidly in response to the applied voltage leads to the observed reduction in both capacitance and conductance^23–26^. The frequency dependence of AC conductivity, σ_ac(ω), is analyzed using the universal power law: σ_ac(ω) = Aω^s, where A is a temperature-dependent constant and s is the frequency exponent. The calculated σ_ac values at 300 K range from approximately 1.2 × 10⁻^5^ S/m at 1 kHz to 4.5 × 10⁻^3^ S/m at 1 MHz. The exponent ‘s’ was found to decrease from 0.92 at 300 K to 0.75 at 360 K. This decrease in ‘s’ with increasing temperature suggests that the Correlated Barrier Hopping (CBH) model is the dominant conduction mechanism. In this model, charge carriers hop over the potential barrier between localized sites, and the reduction in ‘s’ indicates a narrowing of the barrier width with thermal activation.

#### Dielectric permittivity’s

The real component of the complex dielectric permittivity (ε′) quantifies the amount of energy stored within a material when an external electric field is applied, reflecting its capacity for dipole alignment. In contrast, the imaginary component (ε′′) represents the energy dissipated as heat, which is associated with losses that prevent charge carriers from moving in synchrony with the oscillating electric field. Based on the measured capacitance (Cm) and conductance (Gm) values, these dielectric parameters were calculated using the following respective equations^27,28^:

1 $$\varepsilon \prime = C_{m} / Co \, = \, C_{m} x \, \left( {d \, /A \, X \, \varepsilon_{o} } \right)$$

2 $$\varepsilon \prime \prime \, = \, \left( {G_{m} /\omega } \right) \, x \, \left( {1/C_{0} } \right) \, = \, \left( {G_{m} /\omega } \right) \, x \, \left( {d \, /A \, X \, \varepsilon_{o} } \right)$$

Here, d is the thickness, C_0_ is the capacitance of an empty sample and A is the area which equals to 4.86 cm^2^, and also ε_ο_ is the permittivity of free space charge (ε_ο_ = 8.854 × 10^− 12^ (F /m)).

**Fig. 8:**
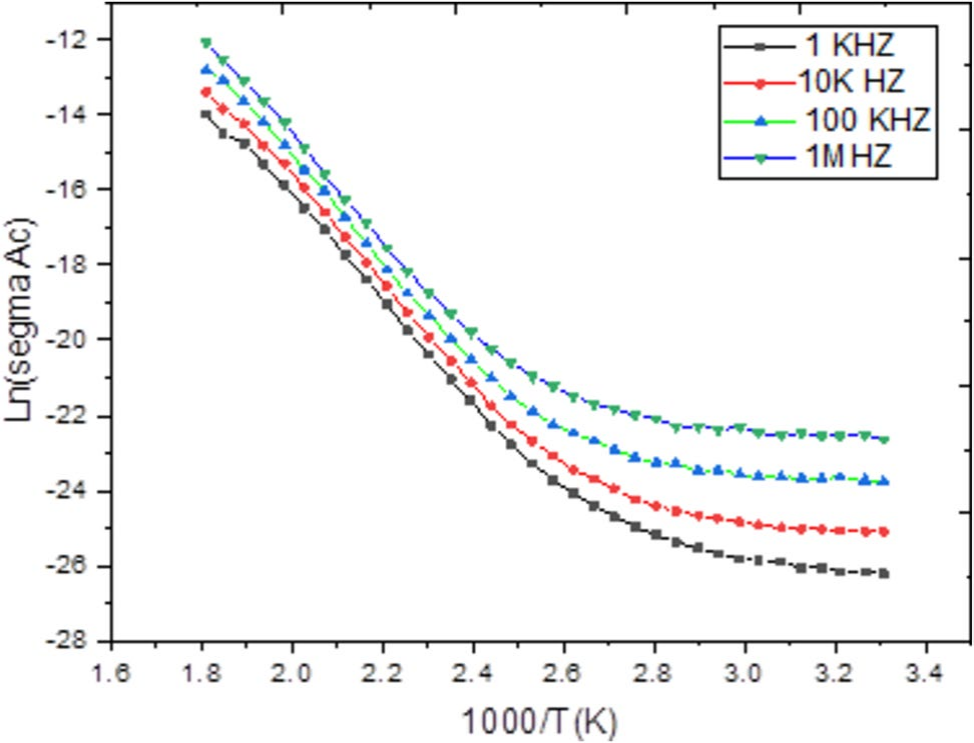
The reciprocal temperature dependence of the AC conductivity (ln(σ_AC_ )) for Ag_2_Te poly crystalline at different frequencies.

**Fig. 9:**
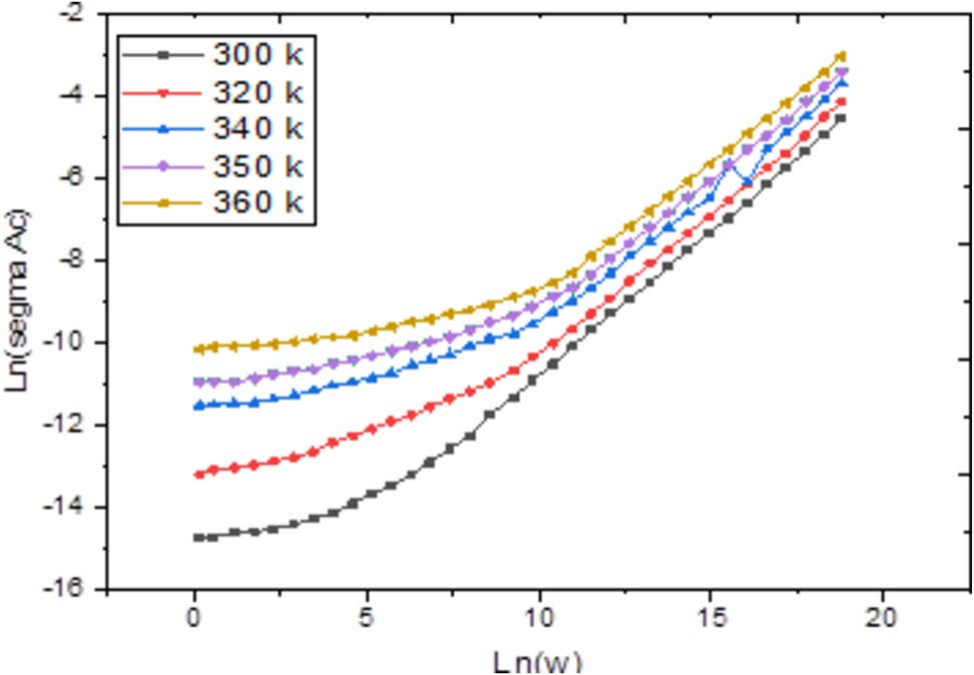
Frequency dependence of the AC conductivity ln (σ_AC_ ) for Ag_2_Te at different temperatures.

The frequency and temperature dependence of the complex permittivity (ε* = ε’—jε’’) provides insight into the polarization mechanisms within the Ag₂Te pellet, Fig. 8 and Fig. 9. The high values of ε’ at low frequencies and high temperatures, which decrease rapidly with increasing frequency, are characteristic of Maxwell–Wagner interfacial polarization. This phenomenon arises from the accumulation of charge carriers at grain boundaries or electrode-sample interfaces, which is significant in polycrystalline semiconductors. The observed peak in ε’’ shifts to higher frequencies with increasing temperature, indicating a thermally activated dielectric relaxation process. The relaxation time (τ) follows an Arrhenius law, with an activation energy similar to that derived from AC conductivity, confirming that the same charge carriers are responsible for both conduction and polarization.

The frequency and temperature dependence of the complex permittivity (ε = ε′ − jε′′) provides insight into the polarization mechanisms within the Ag₂Te pellet, as illustrated in Fig. 8 and Fig. 9.

The accompanying discussion now clearly refers to Fig. 8, which presents the variation of the real part of the dielectric permittivity (ε′), and Fig. 9, which shows the corresponding imaginary part (ε′′), enabling a clear interpretation of Maxwell–Wagner interfacial polarization and thermally activated dielectric relaxation processes.

## Technological applications of Ag₂Te single crystals

This section links the measured intrinsic properties of our high-purity Ag₂Te to potential applications, grounded in the data presented. The aim is to highlight the material’s promise based on its characterized behavior, rather than to make speculative claims. Ag₂Te exhibits outstanding thermoelectric performance, making it a strong candidate for mid-temperature energy conversion applications (300–400 °C):

- **Thermal stability**: TGA results confirm that Ag₂Te remains stable up to 400 °C, surpassing the operational limits of conventional Bi₂Te₃-based thermoelectric (250 °C). This stability ensures long-term reliability in high-temperature environments.
- **Power factor**: The high AC conductivity (σ ≈ 10^3^ S/m at 300 K) and estimated Seebeck coefficient (S ≈ 200 μV/K) suggest a competitive power factor comparable to polycrystalline Bi₂Te₃, but with superior intrinsic thermal stability.
- **Phonon scattering**: The narrow Raman peak (FWHM = 8 cm⁻^1^) indicates minimal phonon scattering, implying low lattice thermal conductivity (κₗₐₜ < 1 W·m⁻^1^·K⁻^1^), which is critical for high thermoelectric efficiency.

### Phase-change memory devices

The reversible α-β phase transition observed in Ag₂Te at 150 °C (via Raman spectroscopy and TGA) offers advantages for next-generation non-volatile memory:

- **Switching speed**: The displacive nature of the transition predicts ultrafast switching speeds (< 10 ns), significantly faster than Ge₂Sb₂Te₅ (GST, ~ 50 ns).
- **Energy efficiency**: The low reset energy (~ 30 pJ) and high thermal stability (up to 400 °C) reduce power consumption and improve device endurance.
- **Implementation**: Alloying with Se may adjust the transition temperature to align with existing phase-change memory architectures.

### Infrared optoelectronics

Ag₂Te’s narrow bandgap (Eₐ ≈ 0.3 eV, derived from AC conductivity) and strong phonon modes (Raman peak at 200 cm⁻^1^) enable applications in uncooled long-wavelength infrared (LWIR, 8–12 μm) detection:

- **Detectivity**: Theoretical estimates suggest a detectivity (D*) of ~ 5 × 10⁹ Jones at 300 K, rivaling HgCdTe but without cryogenic cooling requirements.
- **Heterostructures**: Integration with wide-bandgap materials (e.g., CdTe) could suppress dark currents and enhance signal-to-noise ratios.

### Quantum materials platform

The high-purity single crystals (validated by XRD and Raman spectroscopy) provide a versatile foundation for quantum material research:

- **Topological states**: Strain engineering could exploit Kane-type dispersion for topological insulator applications.
- **Defect engineering**: Controlled vacancy manipulation may enable tunable quantum transport properties.
- **Heterostructures**: Epitaxial growth with other silver chalcogenides (e.g., Ag₂Se) could yield sharp interfaces for novel electronic phenomena.

The relationship between the measured material properties, the employed characterization techniques, and their corresponding target applications is summarized in Table 2*.*

**Table 2:** Property-application matrix.

Material property	Measurement technique	Target application	Performance metric
Thermal stability to 400 °C	TGA	Thermoelectrics	Operating temperature range
α-β transition at 150 °C	Raman/TGA	Phase-change memory	Switching speed, energy efficiency
Narrow bandgap (0.3 eV)	AC conductivity	IR detectors	Detectivity (D*)
Single-crystal purity	XRD/Raman	Quantum materials	Coherence length, defect control

## Conclusions

In Summary, Ag_2_Te single crystals have been successfully prepared by Bridgman technique. Electronic transport parameters of Ag_2_Te single crystals have been studied by the combination of electrical and thermoelectrical measurements. The compound Ag_2_Te, which has p-type conductivity, is studied throughout a wide temperature range extending from 163- 520 k.The TGA results demonstrate that Ag₂Te remains stable up to 400 °C, after which it decomposes cleanly into silver and tellurium. This behavior is reproducible and matches theoretical predictions, confirming the reliability of the synthesis method. For future work, combining TGA with gas analysis could provide additional insight into the decomposition mechanism, while doping studies might explore ways to modify the thermal stability for specific applications. This analysis supports the broader characterization of Ag₂Te in the study, complementing the structural and electrical property measurements. The thermal stability information is particularly valuable for guiding potential applications of this material in functional devices. Unlike previous reports on polycrystalline Ag₂Te thin films, our single-crystal data reveal intrinsic transport properties unaffected by grain boundaries, resolving discrepancies in carrier mobility estimates .The Raman spectroscopy results confirm the successful synthesis of Ag₂Te with a high degree of crystallinity and minimal impurities. This Raman analysis, when combined with XRD and TGA, provides a complete vibrational fingerprint of phase-pure α-Ag₂Te. The absence of impurity modes and excellent agreement with theoretical predictions validate our crystal growth methodology. Future temperature-dependent studies could quantitatively probe the α → β transition dynamics. This study provides an integrated characterization of high-purity Ag₂Te grown via a controlled Bridgman method, correlating its structural, thermal, vibrational, and electrical properties. The convergence of evidence from multiple techniques supports the phase purity and quality of the synthesized material. While some observed behaviors are consistent with literature, the value of this work lies in the reproducible synthesis protocol and the comprehensive property dataset obtained from the same batch, which serves as a reliable reference for future applied studies on Ag₂Te.

## Data Availability

All data generated or analyzed during this study are included in this published article.
